# Common Neural Network for Different Functions: An Investigation of Proactive and Reactive Inhibition

**DOI:** 10.3389/fnbeh.2019.00124

**Published:** 2019-06-07

**Authors:** Fan Zhang, Sunao Iwaki

**Affiliations:** ^1^Graduate School of Comprehensive Human Sciences, University of Tsukuba, Tsukuba, Japan; ^2^Department of Information Technology and Human Factors, National Institute of Advanced Industrial Science and Technology (AIST), Tsukuba, Japan

**Keywords:** proactive inhibition, reactive inhibition, hyperdirect pathway, indirect pathway, dynamic causal models, inferior frontal gyrus, caudate

## Abstract

Successful behavioral inhibition involves both proactive and reactive inhibition, allowing people to prepare for restraining actions, and cancel their actions if the response becomes inappropriate. In the present study, we utilized the stop-signal paradigm to examine whole-brain contrasts and functional connectivity for proactive and reactive inhibition. The results of our functional magnetic resonance imaging (fMRI) data analysis show that the inferior frontal gyrus (IFG), the supplementary motor area (SMA), the subthalamic nucleus (STN), and the primary motor cortex (M1) were activated by both proactive and reactive inhibition. We then created 70 dynamic causal models (DCMs) representing the alternative hypotheses of modulatory effects from proactive and reactive inhibition in the IFG-SMA-STN-M1 network. Bayesian model selection (BMS) showed that causal connectivity from the IFG to the SMA was modulated by both proactive and reactive inhibition. To further investigate the possible brain circuits involved in behavioral control, including proactive inhibitory processes, we compared 13 DCMs representing the alternative hypotheses of proactive modulation in the dorsolateral prefrontal cortex (DLPFC)-caudate-IFG-SMA neural circuits. BMS revealed that the effective connectivity from the caudate to the IFG is modulated only in the proactive inhibition condition but not in the reactive inhibition. Together, our results demonstrate how fronto-basal ganglia pathways are commonly involved in proactive and reactive inhibitory control, with a “longer” pathway (DLPFC-caudate-IFG-SMA-STN-M1) playing a modulatory role in proactive inhibitory control, and a “shorter” pathway (IFG-SMA-STN-M1) involved in reactive inhibition. These results provide causal evidence for the roles of indirect and hyperdirect pathways in mediating proactive and reactive inhibitory control.

## Introduction

The ability to voluntarily suppress actions during inappropriate or even dangerous situations is crucial for human behavior. Previous studies have revealed that successful behavioral control involves both proactive and reactive inhibition (Braver et al., 2007; Jaffard et al., 2007; Aron, 2011; Bari and Robbins, 2013). The former is initiated by prospective cues and involved in response selection (Verbruggen and Logan, 2009; Chikazoe et al., 2009b), while the latter is cue-triggered and occurs when a stop-signal is detected (Eagle et al., 2008; Chambers et al., 2009; Aron et al., 2014). The interactions between proactive and reactive inhibition lead to flexible behavior in a changing environment. Some recent studies have suggested that proactive and reactive inhibition involve shared neural systems (Verbruggen and Logan, 2008; Cunillera et al., 2014; Cai et al., 2016). A model consisting of a multi-step decision process proposed large overlapping in both the frontal and parietal cortex as well as in subcortical brain areas and postulates that the specific interactions between these areas result in withholding or canceling the planned response (Mirabella, 2014). Furthermore, common and unique networks that are centered on frontal networks were proposed to be associated with both proactive and reactive inhibition, *via* a space-independent component analysis (van Belle et al., 2014).

Several paradigms are utilized in behavioral inhibition research. Stop-signal tasks is widely applied in the investigation of the neural mechanisms associated with response inhibition (Logan and Cowan, 1984; Logan et al., 1997). In the classical stop-signal task, participants are required to respond as quickly as possible following the appearance of the “go” stimuli, to withhold their responses in the trials containing “stop” signals, and to change their planned responses in “switch” trials. The stop-signal reaction time (SSRT) is primarily used to estimate the effect of reactive inhibition (Bunge et al., 2002; Band et al., 2003). A modified go/no-go paradigm that incorporates additional cues is usually applied to investigate proactive inhibition. The additional cues in the modified go/no-go task indicate the possibility of upcoming stop-signals, while the SSRT is modified by considering the effect of uncertainty for “go” cues. Recently, a paradigm comparing the reaction times (RTs) and movement times (MTs) of reaching arm movements in different contexts was applied to the investigation of deficits in proactive inhibition in healthy subjects, in Parkinson’s patients, as well as in Tourette and obsessive-compulsive patients (Mirabella et al., 2008, 2013, 2017; Mancini et al., 2018, 2019).

Functional magnetic resonance imaging (fMRI) techniques can be used to identify regions in which increased metabolic activity is induced by reactive inhibition (i.e., no-go > go contrast). The prefrontal cortex (PFC), particularly the inferior frontal gyrus (IFG) and the presupplementary motor area (preSMA)/supplementary motor area (SMA), are critical to reactive inhibition (Simmonds et al., 2008; Chikazoe, 2010; Jahfari et al., 2010; Wardak, 2011; Cunillera et al., 2014; Verbruggen et al., 2014; Rae et al., 2015). An electrocorticographic study of stop-event related potentials has investigated the fronto-temporal lobes of patients with pharmacoresistant epilepsy and found the causal involvement of the premotor area (PMA), the primary cortex (M1), and Brodmann’s area (BA) 9. The study showed that M1 is the destination in the frontal-basal ganglia-thalamic network in a cognitive control task (Mattia et al., 2012). Furthermore, a substantial proportion (30%) of monkey dorsal premotor cortex (PMd) cells produced signals predicting forthcoming actions in a reaching version of the stop-signal task, which suggests that both the M1 and PMd participated in the inhibitory control task (Coxon et al., 2006; Mirabella et al., 2011; Mattia et al., 2013). These areas combine with the basal ganglia to form a network that inhibits the activation of the M1 during reactive inhibition.

In contrast, the metabolic activisty associated with proactive inhibition, which can occur throughout the task, cannot be isolated based on simple neuroimaging contrasts. Previous research has indicated that parts of the reactive inhibition network including the IFG and subthalamic nucleus (STN) are also involved in proactive inhibition (Aron et al., 2014). The application of deep brain stimulation (DBS) to the bilateral STN of patients with Parkinson’s led to shorter SSRT in a stop signal task (van den Wildenberg et al., 2006; Frank et al., 2007; Mirabella et al., 2012a,b), and a similar improvement in appropriate motor strategies that represent proactive inhibitory ability was also found in bilateral DBS of the STN of patients with Parkinson’s (Mirabella et al., 2013). However, no improvement was found with unilateral DBS (Mancini et al., 2019), which is opposed to the hypothesis that the right STN is critical to response inhibition (Aron and Poldrack, 2006; Aron et al., 2014).

The basal ganglia circuits are centrally involved in behavioral control *via* direct (cortico-striato-nigral) and indirect (cortico-striato-pallido-subthalamonigral) pathways (Alexander et al., 1986; Albin et al., 1989; DeLong, 1990; Aron et al., 2007a,b). Both pathways receive cortical input, with a dissociation between facilitation and suppression of the initiated activation. The former is hypothesized to activate the motor cortex and the latter is considered to suppress movement. For the direct pathway, the cortical activity is transmitted to the striatum, then the activated striatum inhibits the activity of the globus pallidus internalis (GPi), which leads to the disinhibition of the thalamus and then to the generation of movement. In contrast, the activated striatum inhibits the globus pallidus externalis (GPe) *via* the indirect pathway, which inhibits the activity of the thalamus and leads to movement inhibition. Another pathway, the hyper-direct pathway, which is also involved in motion information processing (Nambu et al., 2000, 2002; Baker et al., 2010; Dunovan et al., 2015), bypasses the striatum and connects the cortex and STN directly, and then activates the GPi to inhibit the activity of the thalamus in a faster way. Recently, a study related to cortical-basal ganglia networks provided a new hypothesis of action suppression: the action is paused *via* a subthalamic-nigral pathway in the preparation step and canceled through arkypallidal GABAergic projections (Mallet et al., 2016).

We hypothesized that there are overlapping regions in the neural systems involved in proactive and reactive inhibitory processes and that the effective connectivities between the relevant brain regions are modulated differently in those processes. In this study, we used a special neuroimaging contrast to isolate fMRI activity associated with proactive inhibition *via* the stop-signal paradigm, in order to identify the cortical and subcortical areas involved in proactive and reactive inhibition. We then incorporated the identified regions of interest (ROIs) into dynamic causal models (DCMs) of proactive and reactive inhibition. Bayesian model selection (BMS) was applied to investigate the effective connections associated with each type of inhibition.

## Materials and Methods

### Participants

Twenty healthy right-handed adults (age, mean ± SD, 21.75 ± 2.57; range: 19–31 years; 11 males), excluding 10 participants with excessive head movement in the MRI scanner defined as translational or rotational displacement greater than 2.5 mm in any direction, performed a stop-signal task during fMRI scanning. All participants were recruited from the University of Tsukuba as paid volunteers, had normal or corrected-to-normal vision and provided written informed consent prior to the experiment. No participant was taking medicine during the experiment. The present study was approved by the Institutional Review Board (IRB) of the National Institute of Advanced Industrial Science and Technology (approval number: 2014-481) and all participants gave informed consent prior to participation.

### Stop-Signal Task

**Figure 1:**
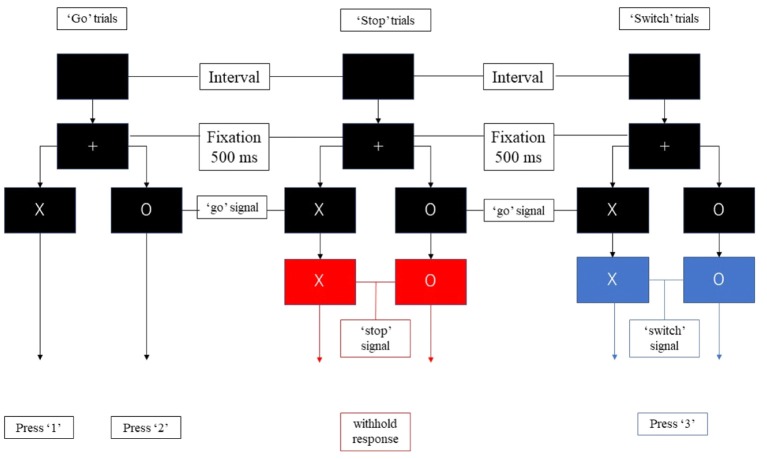
The components of the stop-signal task. Participants were required to press “1” or “2” as quickly as possible following the appearance of the stimulus unless the color of the background changed to red or blue. They were required to withhold response when the background turned red, and to press “3” when the background turned blue.

In the present study, we divided the “go,” “stop,” and “switch” trials into several substages to isolate proactive and reactive inhibition (Figure 1). Unlike previous studies regarding proactive inhibition, we did not use additional cues to indicate “certain go” trials. All trials of the present study remained “uncertain” at the initial stage. During each trial, a fixation cross appeared on a black background for 500 ms, then the point of fixation cross was replaced by the initial character (“X” or “O”) for 1,500 ms. Participants were required to press “1” on the button-box if the stimulus was “X” and “2” if “O” appeared unless the background color change. If the background color changed to blue, participants need to switch their response to press “3.” If the background color change to red, participants were instructed to withhold their response regardless of the current initial character. The duration between the appearance of initial character and the change of background color is 500 ms. Thus, participants were required to withhold their planned response and wait for any possible upcoming cue to avoid an incorrect response when the initial character (“X” or “O”) appeared. Participants had to totally abort the responses that were already in progress if the background changed to red or switch their response to press “3” if the background changed to blue. The proactive component was thus present in all trials (“go,” “stop,” and “switch” trials), and the reactive component was present in successful “stop” and “switch” trials.

Each run consisted of 40 “go” trials, 10 “stop” trials, and 10 “switch” trials. An equal distribution of the characters “X” and “O” was ensured across trials, in random order. Each participant needs to complete three runs.

We applied paired *t*-test on mean RTs for “go” and “switch” trials to test if there were significant difference between them. Because the fixed stop-signal delay (SSD) was used in the current procedure, we estimated SSRT with integration method (Logan and Cowan, 1984) by subtracting SSD from the finishing time that is determined by the distribution of no-signal go RTs.

### fMRI Data Acquisition

All fMRI scans were obtained using a 3-Tesla scanner (Ingenia 3T, Philips, Netherlands) at the Department of Information Technology and Human Factors, AIST (Tsukuba, Japan). Each participant’s head was fixed using foam padding to reduce head movement. Single-shot echo-planar imaging (EPI) sequences were used to acquire functional images. EPI parameters were as follows: repetition time (TR) = 2,000 ms; echo time (TE) = 35 ms; flip angle = 90°, 31 ascending slices, thickness = 3.7 mm.

### Data Processing

The SPM12 software toolbox^1^ and Matlab 2015b were used for the analysis of fMRI data and for the creation of the DCMs. All coordinates are reported in standard Montreal Neurological Institute (MNI) space and were labeled using the Anatomical Automatic Labeling (AAL) toolbox in SPM12 (Brett et al., 2002; Tzourio-Mazoyer et al., 2002). Successful “go” trials were regarded as those in which the participant selected the appropriate button following the waiting period. Successful “stop” and “switch” trials were regarded as those in which the participant withheld appropriate responses until the subsequent signals appeared.

**Figure 2:**
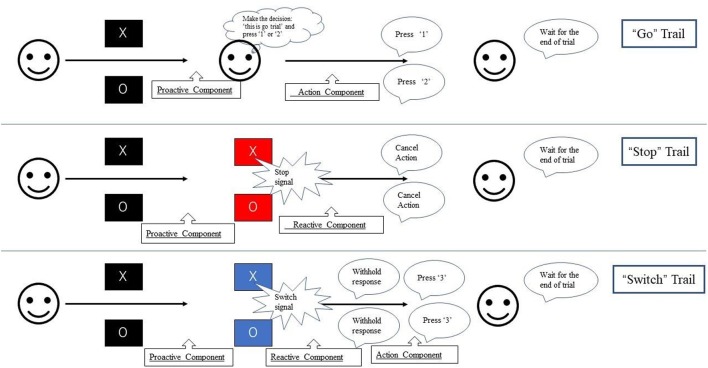
Experimental paradigm used in this study. During each trial, the fixation cross was replaced by the initial character (“X” or “O”) for 1,500 ms. Participants were required to press “1” on the button-box if the stimulus was “X” and “2” if “O” appeared unless the background color change. If the background color changed to blue, participants need to switch their response to press “3.” If the background color change to red, participants were instructed to withhold their response regardless of the current initial character. The duration between the appearance of initial character and the change of background color is 500 ms. Thus, participants were required to withhold their planned response and wait for any possible upcoming cue to avoid an incorrect response when the initial character (“X” or “O”) appeared. Participants had to totally abort the responses that were already in progress if the background changed to red or switch their response to press “3” if the background changed to blue.

We divided the trials into several components (Figure 2). All trials of the present study remained “uncertain” at the initial stage. When the initial character (“X” or “O”) appeared, participants were required to withhold their planned responses and wait for any possible upcoming cue to avoid an incorrect response. Thus, the proactive inhibitory component appeared at the beginning of all trials. The action component was involved when participants figured out the “go” trial and pressed the corresponding button. For both “stop” and “switch” trials, participants needed to cancel the planned action that resulted in reactive inhibitory component. The difference is in “switch” trial, participants needed to press the alternative button, which led to subsequent action component. Based on the above reasons, the components in the “go” trials included proactive inhibitory component and an action component. The “stop” trials were subdivided into a proactive and a reactive component, and the “switch” trials consisted of a proactive, a reactive, and an action component.

Based on this approach, reactive inhibition was analyzed by comparing the successful “switch” trials to successful “go” trials. We did not apply the classical comparison that used for race model between successful “stop” trials (proactive inhibitory component + reactive inhibitory component) and successful “go” trials (proactive inhibitory component + action component) because the result cannot be explained by isolated reactive inhibitory component. Proactive inhibition was isolated by the conjunction of all successful trials (“go,” “stop,” “switch”). We used a general linear model for first-level event-related analysis in each participant. Events (successful “go,” successful “stop,” successful “switch”) were modeled after a duration of 0.5 s from trial onset. A second-level SPM analysis used contrasts from the first level with one-sample tests to investigate the group-level activation. A peak-level false discovery rate (FDR) at *p* < 0.05 was applied to correct for multiple comparisons.

### Dynamic Causal Modeling for Comparing Proactive and Reactive Modulation

We used DCM12 (Friston et al., 2003) for the analysis of effective connectivity between the prior selected set of brain regions. fMRI-based DCM is a deterministic model of neural dynamics that describes how neural activity and interactions generate the hemodynamic BOLD response. The effective connectivity between brain regions or nodes was estimated by three matrices: the endogenous connectivity between nodes (A matrix), the modulation effects on the connection during the special experimental conditions (B matrix), and the driving inputs that influence the connectivity to other nodes (C matrix). If there were connections modulated by other regions (nonlinear connectivity effects), the parameters were modeled as an additional matrix (D matrix).

We defined MNI coordinates of the ROIs for DCM analysis that met all of the following criteria: (1) the coordinate of the spherical ROIs should show significant activations in both proactive and reactive contrasts with a cluster-based FDR at *p* < 0.05 in the second-level SPM analysis; and (2) the regions have been reported to be involved in behavioral control in previous research. We constructed 70 DCM models on four regions: the right IFG (*x* = 48, *y* = 16, *z* = 30), the left SMA (*x* = −6, *y* = −10, *z* = 50), the STN (*x* = −10, *y* = −14, *z* = −6), and the M1 (*x* = −30, *y* = −12, *z* = 66).

**Figure 3:**
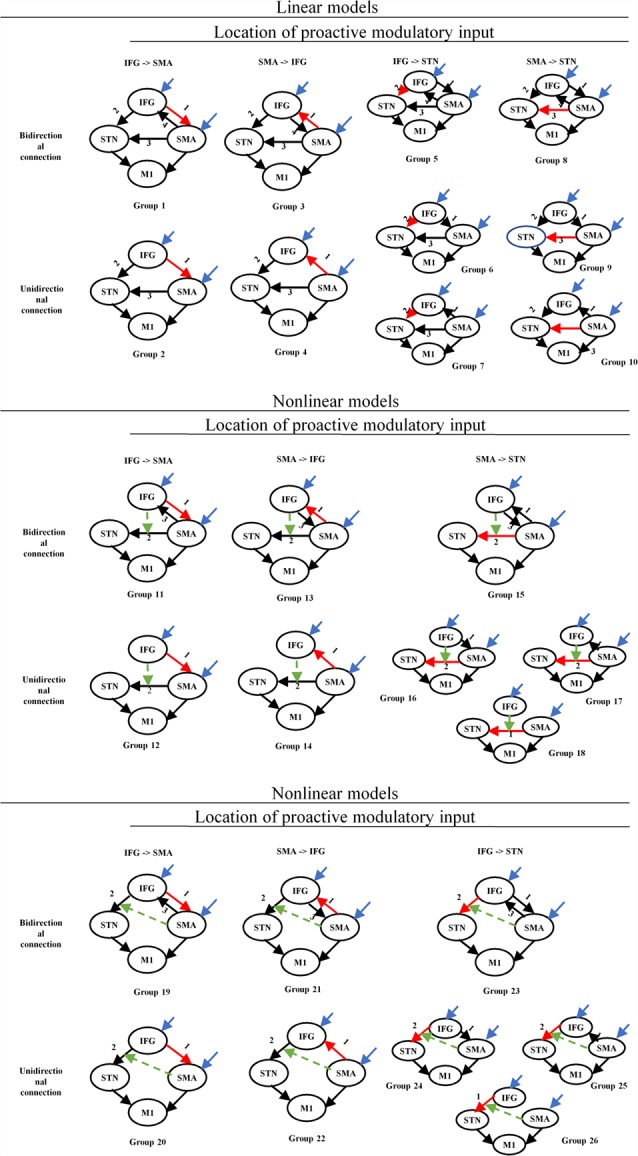
Structure of the DCM families tested for proactive and reactive inhibition. Red arrows represent the location associated with the proactive modulatory input. Dotted arrows represent the nonlinear modulation. The different numbers represent the different locations related to the reactive modulatory input. DCM, dynamic causal modeling; M1, primary motor cortex; IFG, inferior frontal gyrus; DLPFC, dorsolateral prefrontal cortex; STN, subthalamic nucleus; SMA, supplementary motor area.

The set of 70 DCM models was divided into two groups: a linear and a nonlinear group. Each group was divided into several sub-groups based on the location of the proactive modulatory input (IFG to SMA, SMA to IFG, IFG to STN, SMA to STN) and the connectivity between the IFG and the SMA (bidirectional, unidirectional, no connection; Figure 3). Average self-connections were applied to all nodes. The STN was connected with the IFG and the SMA directly or indirectly.

The nodes, which receive driving input, are the regions in the model that first experience the neural changes caused by the manipulations of the experimental conditions. The modulatory inputs, which represent the specific experimental factor, realize the modulation by influencing the intrinsic connections in the network (Penny et al., 2004). Thus, the experimental conditions we chose as modulatory inputs should include the specific experimental factor and avoid the other factors that may drive the network activity in different modulatory effects. Both experimental conditions, that is, “stop” and “switch” trials, include the reactive inhibitory component, and for this reason, we selected all “go” trials (correct “go,” incorrect “go”) as proactive modulators to separate proactive from reactive modulation. We considered the frontal regions (IFG and SMA) as nodes receiving driving inputs across all models. The modulatory inputs include proactive modulatory inputs and reactive modulatory inputs. We applied the modulatory inputs in the fronto-STN connections (IFG to SMA, SMA to IFG, IFG to STN, SMA to STN). All trials (“go,” “stop,” “switch”) were chosen as driving inputs, which represent the extrinsic influences on the IFG-SMA-STN-M1 network. To separate proactive from reactive modulation, we selected all “go” trials (correct “go,” incorrect “go”) as proactive modulator. The reactive modulator was acquired by selecting “stop” and “switch” trials in which participants provided appropriate responses following the appearance of the signal.

We defined group peak coordinates from the second-level group analysis of proactive and reactive contrasts, combined with the AAL atlas implemented in the SPM toolbox. All trials were used for extracting the first eigenvariate of the BOLD time series for STN and M1, and the conjunction contrast of proactive and reactive modulators for IFG and SMA. All first eigenvariates were adjusted for the *F*-test of effects of interest. To extract the time series from the ROIs for each participant, we combined the local maximum close to the group peak and extracted the eigenvariate from a 5-mm sphere.

## Results

### Behavioral Data

There were significant differences in mean RTs between “go” trials (mean ± SD, 963 ± 74 ms, range: 836–1092 ms) and “switch” trials (mean ± SD, 1,120 ± 87 ms, range: 948–1,350 ms; *p* < 0.0001). For “go, ” “stop, ” and “switch” trials, mean accuracy was 0.890 (SD: 0.117) and 0.853 (SD: 0.165), respectively. The mean SSRT was 454 ms (range: 304–737 ms, SD: 96 ms).

### Group-Level Activations

**Figure 4:**
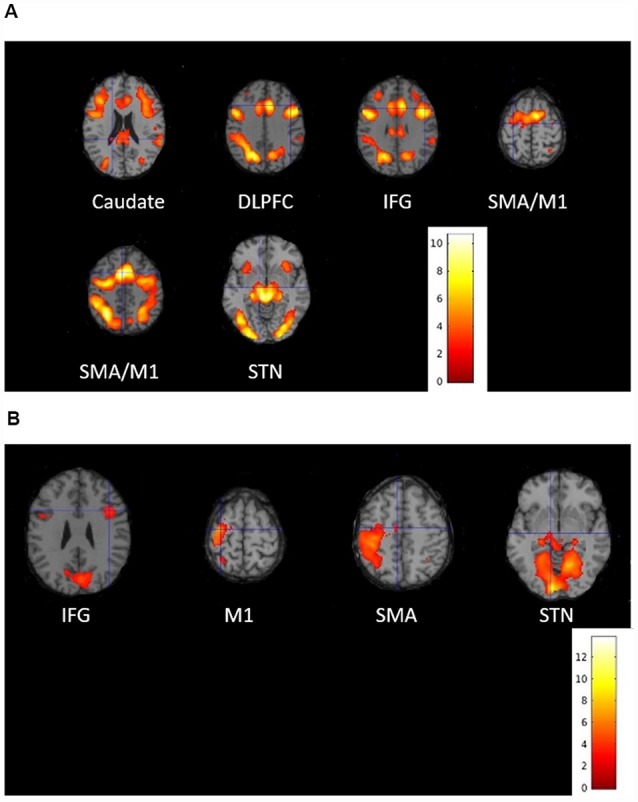
Activated brain regions associated with **(A)** proactive inhibition and **(B)** reactive inhibition during the stop-signal functional magnetic resonance imaging (fMRI) experiment. Group-level statistical maps were calculated **(A)** as conjunction of all successful “go,” “stop” and “switch” trials for proactive inhibition and **(B)** as a contrast between successful “switch” trials and successful “go” trials for reactive inhibition. The results were thresholded at peak-level false discovery rate (FDR)-corrected significance of *p* < 0.05.

Activity associated with proactive inhibition obtained by the conjunction of all successful “go,” “stop” and “switch” trials, was significant in the visual cortex, dorsolateral prefrontal cortex (DLPFC), caudate, SMA, IFG, STN and M1 of both hemisphere (Figure 4A; Supplementary Table 1). As for the reactive inhibition, we found activation in the right IFG, the left SMA, left M1, as well as bilateral activation of STN (Figure 4B; Supplementary Table 2). Because the volume of STN is about 240 mm^3^ in humans that means the activation of STN is only eight voxels (Hardman et al., 2002; Hamani et al., 2004), we chose the coordinate of the STN described in Forstmann et al. (2012) as a reference to confirm the STN activated significantly.

### DCM Network Analysis for Comparing Proactive and Reactive Modulation

**Figure 5:**
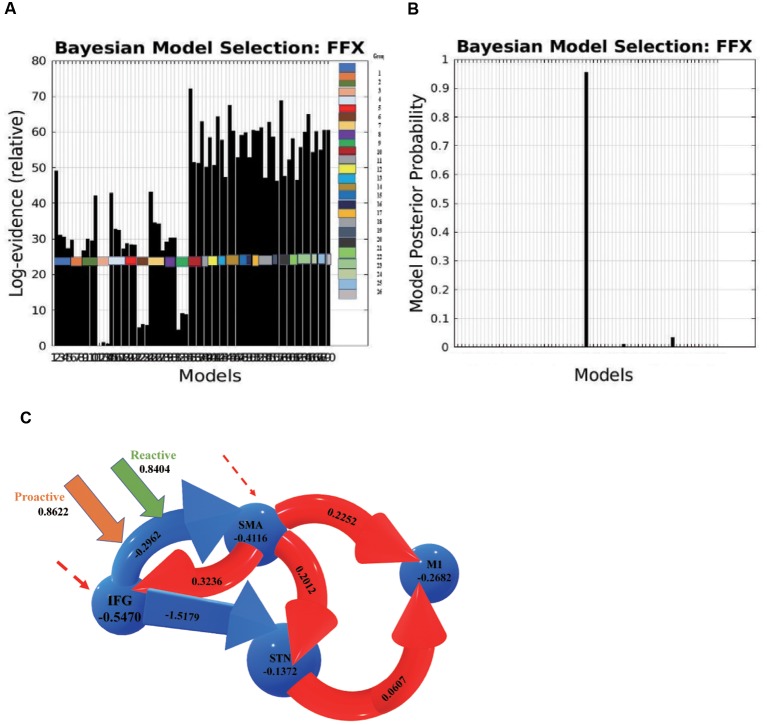
**(A)** Log evidence and **(B)** model posterior probability to compare DCM model families, and **(C)** the most possible model selected based on Bayesian model selection (BMS) denoting connectivity coefficients for the comparison between proactive and reactive modulatory inputs. The connections in red represent increased excitatory connectivity, while blue connections represent decreased inhibitory connectivity. The dotted lines represent the driving inputs.

We investigated 70 DCMs representing the alternative hypotheses of modulatory effects from proactive and reactive inhibition. BMS with fixed-effect analysis (FFX) provided strong evidence for one model being optimal, above all other models (Figure 5).

Two parameters were measured in the BMS to compare the optimal architecture of the models: relative log-evidence and posterior probability. The former corresponds to the balance between accuracy and complexity of the model, while the latter represents the probability that the specific model provides the best explanation for all participants.

DCM estimates the observed BOLD responses and models the effects of neuro-vascular processes and spectral noise at different levels. The parameters represent the rate of change in activity in one region that influences the activity in another region, and the effective connectivity was thus expressed in Hz.

The average connectivity between two regions represents how rapidly activity (per second) in one region influences the activity in another region (Friston et al., 2003; Penny et al., 2004; Almgren et al., 2018). A positive value represents an excitatory influence from the source region on another region, while a negative value represents an inhibitory influence. Likewise, modulatory effects on a region or connection indicate an increase or decrease in average activity or connectivity.

In this optimal nonlinear model, the right IFG modulates the connection between the left SMA and the left STN, and the connection from the IFG to the SMA is modulated by both proactive (modulation effect = 0.8622 HZ) and reactive modulatory inputs (modulation effect = 0.8404 HZ). The results indicate that when people “prepare to cancel” and then “cancel” a planned response successfully, both proactive and reactive modulation influence on the effective connection from the IFG to the SMA. The IFG inhibits the activity in the SMA, and the decreased activity in the SMA influences the subsequent areas *via* a causal relationship and then increases the excitatory influence of the STN on the M1.

### DCM Network Analysis for Proactive Inhibition

To further investigate the possible brain circuits involved in the implementation of behavioral control including proactive and reactive inhibitory processes, we added extra regions to the DCM models for proactive inhibition. The regions of interests were selected based on the same criteria as the previous DCM models, i.e., (1) these regions should show significant activation in proactive contrasts with a cluster-based FDR at *p* < 0.05 in the second-level SPM analysis; and (2) the regions have been reported to be involved in behavioral control in previous research. We constructed 13 DCM models including four ROIs: the right IFG, the DLPFC (*x* = 44, *y* = 20, *z* = 36), the left SMA, and the caudate (*x* = **−**16, *y* = **−**30, *z* = 24). As previous results showed that the modulatory effects were related to the connection from the IFG to the SMA, this modulatory effect should be a direct or indirect effect from other effective connections that end in the IFG.

We limited our model for proactive inhibition to the four regions based on the following reasons: (1) the previous studies suggested the top-down control for proactive inhibitory control (Jaffard et al., 2007, 2008; Criaud et al., 2012). Based on the result from the DCM network analysis for comparing proactive and reactive modulation, it revealed that the modulatory effects happened on the effective connectivity from IFG to SMA. Thus, it is reasonable to believe that the proactive modulation would not firstly act on the downstream connections that causally follows the effective connection from IFG to SMA; and (2) the increasing number of nodes in DCM will lead to over much numbers of free parameters that require exponentially increasing computational time. Furthermore, the conditional dependencies among these parameters will also enhance that influence the reliability of explanation for DCM model (Daunizeau et al., 2011). Based on these reasons, we think our model with four regions DLPFC-caudate-IFG-SMA is sufficient to investigate the proactive modulation.

Since converging evidence indicates that the prefrontal areas project to the STN, in the DCM analysis of proactive inhibition, the DLPFC-IFG-SMA-caudate is the minimum and effective set required to test hypotheses. We have found reactive modulatory effects on effective connection from IFG to SMA in the previous step, so there are two possibilities for the “real” neural underpinning of reactive modulation: (1) the IFG-SMA is the real effective connectivity that is modulated by reactive inhibition; and (2) the real effective connectivity is the other connectivity in prefrontal-STN network. The modulatory effect was transferred to the effective connectivity IFG to SMA and observed in the IFG-SMA-STN-M1 DCM model. Based on the result from whole brain contrast related to reactive inhibition, there are no other activations in frontal areas involved in inhibitory control and prefrontal-STN connections, which means no other effective connectivity responds to reactive inhibition, so the IFG-SMA is the real effective connectivity that modulated by reactive inhibition.

**Figure 6:**
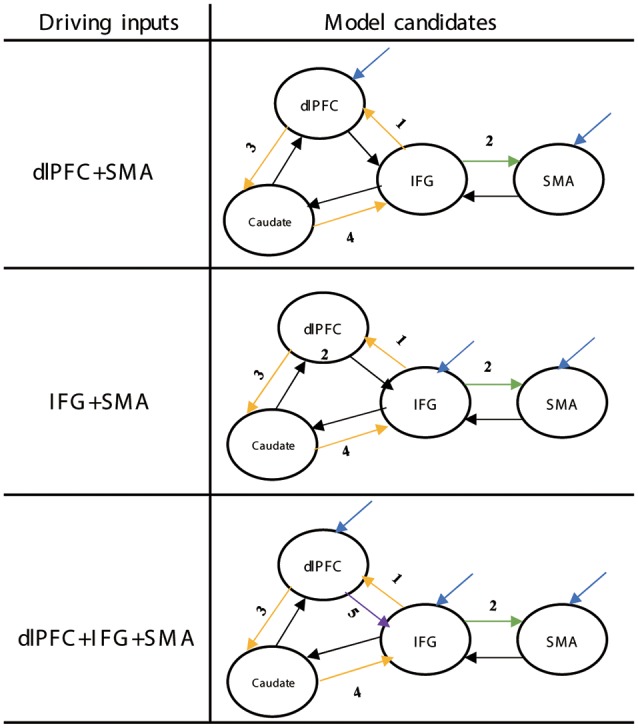
Structure of the DCM families tested for proactive inhibition. The yellow arrows show the direction of loops that represent indirect modulation from other effective connections that end in the IFG. The different numbers represent the different locations related to the proactive modulatory input. DCM, dynamic causal modeling; M1, primary motor cortex; IFG, inferior frontal gyrus; DLPFC, dorsolateral prefrontal cortex; STN, subthalamic nucleus; SMA, supplementary motor area.

The set of 13 DCM models was divided into three groups based on whether the DLPFC receives driving inputs (driving inputs were applied to DLPFC + SMA, IFG + SMA, and DLPFC + IFG + SMA). The location of proactive modulatory inputs varied in the bidirectional connections in the DLPFC-IFG-SMA-caudate network, and locations were chosen based on the requirement that the indirect modulatory effects from other effective connections should end in the IFG. Average self-connections were applied to all nodes. The interconnectivities between nodes are bidirectional (Figure 6). We considered the frontal regions (DLPFC and SMA, IFG and SMA, DLPFC/IFG/SMA) as nodes receiving driving inputs across all models. The modulatory inputs are only proactive inputs. We applied modulatory inputs to the effective connections in the DLPFC-caudate-IFG-SMA network (IFG-DLPFC, DLPFC-IFG, IFG-SMA, DLPFC-caudate, caudate-IFG).

We extracted the first eigenvariate of the BOLD time series from two regions of interests, the DLPFC and the caudate. All time series were adjusted for the *F*-test of effects of interest. To extract the time series from the ROIs for each participant, we combined the local maximum close to the group peak and extracted the eigenvariate from a 5-mm sphere.

**Figure 7:**
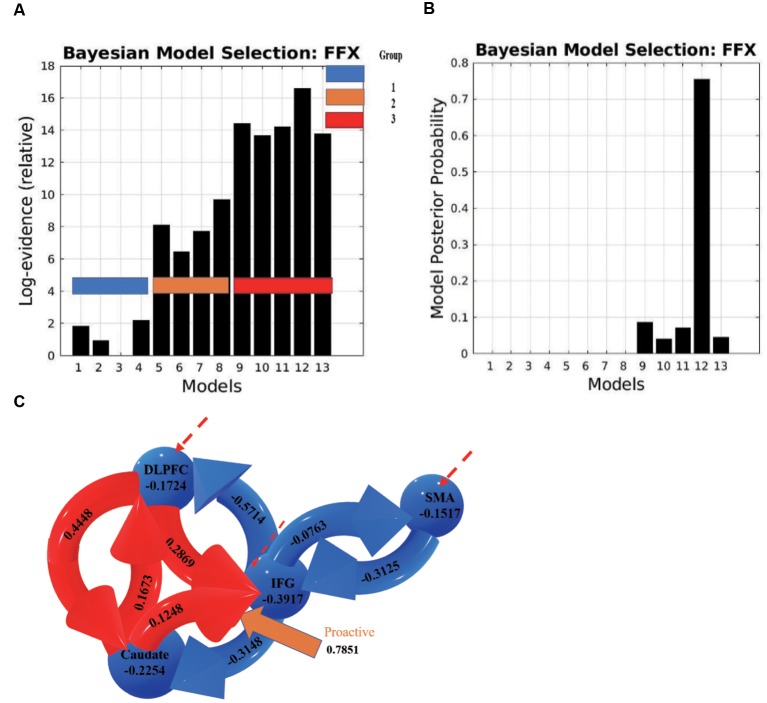
**(A)** Log evidence and **(B)** model posterior probability to compare DCM model families, and **(C)** the most possible model selected based on BMS denoting connectivity coefficients for the DCMs with proactive modulatory inputs. The connections in red represent increased excitatory connectivity, and blue connections represent decreased inhibitory connectivity. Red dotted lines represent the driving inputs.

The results of BMS (FFX) indicated that there was one model that was superior to all other models (Figure 7). In this model, the connection from the caudate to the IFG was associated with the proactive modulation (modulation effect = 0.7851). The results indicate that when people prepare for a possible upcoming stop-signal, the caudate increases its excitatory influence on the IFG, which leads to an inhibitory influence of the IFG on the SMA.

## Discussion

In the present study, we used fMRI data acquired during a stop-signal paradigm task to identify the cortical and subcortical areas involved in proactive and reactive inhibitory processes. To evaluate the modulatory effects of proactive and reactive inhibition on the effective connections between these areas, we first conducted a DCM analysis where 70 DCM models were compared. The results indicate that the increasing activity in effective connectivity from the left SMA to the left STN was modulated by the right IFG, and the decreasing activity in effective connectivity from the right IFG to the left SMA was modulated by both proactive and reactive modulatory effects. We further investigated an alternative hypothesis with 13 additional DCM models in which the causal connection from/to the right DLPFC and the left caudate were considered for proactive inhibition. The results of the additional DCM model comparison show that the increased activity of the effective connection from the left caudate to the right IFG was modulated by proactive modulatory control, which resulted in the inhibitory effects in the connections from the right IFG to the left SMA in the comparison between proactive and reactive inhibitory control.

The fronto-basal ganglia pathways have been proposed to support motor control *via* hyperdirect and indirect pathways. Previous studies suggested that the right IFG and left SMA are critical regions in inhibitory control (Aron et al., 2004; Chambers et al., 2006; Aron et al., 2014). However, it is still difficult to assign a very specific role to most of these regions during the execution of complex cognitive functions (Mirabella, 2014; Hampshire, 2015). For example, some studies revealed that the right IFG acts as a monitor of unexpected stimuli (Corbetta and Shulman, 2002), and others show that it is involved in the suppression of memories (Benoit and Anderson, 2012).

A significant overlap was reported in the brain systems underlying proactive and reactive inhibition *via* modified stop signal task or extra information about the probability of the occurrence of stop signals, and showed that the right IFG, the pre-SMA/SMA, and part of the basal ganglia circuit (striatum) are involved in both proactive and reactive inhibition (Chikazoe et al., 2009a; Swann et al., 2012; Aron et al., 2014). Furthermore, the recent research reveals that the neural network involved in goal-directed cognitive control is very extensive. The multi-step decision process model proposed that many areas such as the DLPFC, PMd, and M1 are also involved in goal-directed behavior (Kenner et al., 2010; Mirabella, 2014). However, previous studies were unable to determine the interactions that exist between these regions or to describe how the brain can “identify” the different kinds of inhibitory control.

We used DCMs to demonstrate which connections in the common network contribute to proactive and reactive inhibitory control. The DCMs also provided us with information regarding the directions and excitatory or inhibitory modulatory effects of these connections. Our results reveal that the effective connection from the IFG to the SMA is associated with both proactive and reactive modulatory effects, which is in line with previous neurophysiology and neuroimaging evidence showing that the IFG is connected with the SMA. Our results further show that in the most likely model, both proactive and reactive inhibition decreased the excitatory influence from the IFG to the SMA and inhibited the activity of M1.

The results of our further investigation of proactive inhibition show that the neural underpinning of proactive modulation is the effective connection from the right DLPFC *via* the left caudate to the right IFG, while the subsequent effect of transmission is reflected in the effective connection from the IFG to the SMA in a common network. The brain thus uses the DLPFC-caudate-IFG-SMA-STN-M1 pathway to implement proactive modulation. These results support the prior hypothesis that basal ganglia circuits are involved in proactive and reactive inhibition, which suggested that a hyperdirect pathway that allows for faster behavioral control than the direct and indirect pathways, by bypassing the striatum, is involved in reactive inhibition. The indirect pathway is functionally similar to the hyperdirect pathway but transfers the modulatory effects through the striatum.

Our present study also provides new evidence for the functions of the right IFG. Although previous studies have extensively investigated the role of the right IFG in response inhibition, findings remain controversial. It remains, for example, unclear whether the right IFG is associated with modulatory inhibition or with the more general detection of salient or task-relevant cues. Furthermore, the right IFG is considered to expedite inhibition processes *via* the pathway from the pre-SMA/SMA to subcortical regions, based on findings from neuroimaging and transcranial magnetic stimulation studies (Aron et al., 2003; Chambers et al., 2006). However, electrophysiological studies have indicated that activity in the pre-SMA/SMA can precede activity in the IFG during response inhibition (Swann et al., 2012). Our results indicate that the right IFG acts as a driving input during reactive inhibition, supporting the notion that this region plays a key role in detecting task-relevant cues. These results, therefore, suggest that the IFG is related to attentional switching. These results are consistent with previous findings showing that right-IFG activity was higher when infrequent stimuli were detected, indicating that this region does not belong to a unique network involved in inhibitory control (Hampshire et al., 2010; Erika-Florence et al., 2014).

Our study furthermore provides evidence for the functions of the right DLPFC. Comparative studies involving both humans and non-human primates have concluded that the PFC is a crucial neural substrate of cognitive control (Servan-Schreiber et al., 1996; Assad et al., 1998; Cohen et al., 1999; Jahfari et al., 2012; Smittenaar et al., 2013). Recent studies have also revealed that the DLPFC plays a central role in the maintenance of goals and rules for action (Watanabe, 1990, 1992; Asaad et al., 2000). Additional studies have demonstrated that the DLPFC monitors environmental cues to develop appropriate response strategies (Ragozzino, 2007; Hikosaka and Isoda, 2010). These findings are consistent with our result that the DLPFC acts as a driving input during proactive inhibition.

Previous tract-tracing studies in monkeys and diffusion tensor imaging studies in humans have indicated that the DLPFC is connected with the caudate (Parent, 1990; Parent and Hazrati, 1995; Lehéricy et al., 2004), and that the DLPFC-caudate circuit is involved in selective inhibition *via* the indirect pathway (Mink, 1996; Jahfari et al., 2011). Previous animal, clinical, and neuroimaging studies have provided extensive evidence that the caudate was involved in the selection of appropriate response based on the assessment of the outcomes, and the studies of patients with impairments in the caudate nucleus also showed the deficit in goal-directed tasks (Hazrati and Parent, 1992; Cai et al., 2011; Bryden et al., 2012; Majid et al., 2013). Our results indicate that the left caudate is related to modulatory input, consistent with the findings of previous studies reporting that the caudate nucleus contributes to behavior through the selection of appropriate sub-goals.

In the current study, we used fMRI to investigate inhibitory behavior. The limited temporal resolution of fMRI may result in limited scope in the DCM analysis. Therefore, multiple methods such as electroencephalography (EEG) or ECoG, which have better temporal resolution, need to be considered in future research. Further, we had to exclude participants with excessive head movement, and the reduced number of effective participants might have caused us to miss some regions with significant activations.

## Conclusion

We show that the effective connection from the IFG to the SMA is associated with reactive inhibition, while the effective connection from the caudate to the IFG is associated with proactive inhibition. The indirect DLPFC-caudate-IFG-SMA-STN-M1 pathway is involved in the implementation of proactive modulation, while the hyperdirect pathway that bypasses the striatum contributes to reactive inhibition. The function of the IFG is more related to attention control, and the caudate more likely acts as a “gate” between proactive and reactive inhibition.

## Ethics Statement

The present study was approved by the Institutional Review Board (IRB) of the National Institute of Advanced Industrial Science and Technology (approval number: 2014-481) and all participants gave informed consent prior to participation.

## Author Contributions

FZ and SI conceived and designed the experiments, planned and carried out the experiments. FZ performed the data analysis, interpreted the results, and wrote the manuscript with critical feedback from SI.

## Conflict of Interest Statement

The authors declare that the research was conducted in the absence of any commercial or financial relationships that could be construed as a potential conflict of interest.

## Footnotes

^1^http://www.fil.ion.ucl.ac.uk/spm/

## Abbreviations

STN: Subthalamic nucleus
IFG: Inferior frontal gyrus
M1: Primary motor cortex
SMA: Supplementary motor area
PFC: Prefrontal cortex
RIFC: Right inferior frontal cortex
DLPFC: Dorsolateral prefrontal cortex
DCM: Dynamic causal model
BMS: Bayesian model selection
SSRT: Stop-signal reaction time
RT: Reaction time
MT: Movement time
fMRI: Functional magnetic resonance imaging
EEG: electroencephalography
PMA: Premotor area
BA: Brodmann’s area
PMd: Dorsal premotor cortex
DBS: Deep brain stimulation
GPi: Globus pallidus internalis
GPe: Globus pallidus externalis
ROI: Region of interest
EPI: Echo-planar imaging
TR: Repetition time
TE: Echo time
MNI: Montreal Neurological Institute
AAL: Anatomical Automatic Labeling
FDR: False discovery rate
FFX: Fixed-effect analysis.
