# Supplementary material for: Common Neural Network for Different Functions: An Investigation of Proactive and Reactive Inhibition
Source: Front Behav Neurosci. 2019 Jun 7;13:124. doi: 10.3389/fnbeh.2019.00124 (PMC6568210; doi:10.3389/fnbeh.2019.00124)
Supplement: Supplementary file 1 [file Table_1.DOCX]

Supplementary Material

# Supplementary Table 1. Activated brain regions associated with proactive inhibition during the stop-signal fMRI experiment. Group-level statistical map was calculated as conjunction of all successful “go”, “stop” and “switch” trials. The results were thresholded at peak-level FDR-corrected significance of p < 0.05.

| Regions | MNI coordinates | | | Peak-level | | |
| --- | --- | --- | --- | --- | --- | --- |
|  | x (mm) | y (mm) | z (mm) | T | Z | q_FDR-corr_ |
| Supplementary motor area_Left | -4 | 12 | 48 | 10.56 | 7.82 | 0.000 |
| Paracentral Lobule_Right | 12 | -32 | 42 | 2.69 | 2.60 | 0.024 |
| Primary motor cortex_Left | -16 | -16 | 58 | 2.37 | 2.30 | 0.048 |
| Caudate_Left | -18 | -36 | 22 | 2.99 | 2.87 | 0.012 |
| Rolandic operculum_Right | 54 | -18 | 22 | 2.82 | 2.71 | 0.018 |
| Occipital corext_Left | -18 | -64 | 36 | 8.91 | 7.02 | 0.000 |
|  | -34 | -64 | -16 | 8.82 | 6.98 | 0.000 |
| Parietal_Inf_Left | -30 | -56 | 46 | 10.63 | Inf | 0.000 |
| Lingual_Left | -4 | -30 | -6 | 10.24 | 7.68 | 0.000 |
| Thalamus_Right | 6 | -26 | -8 | 10.06 | 7.60 | 0.000 |
| Cingulate_Mid_Left | -6 | -26 | 28 | 8.39 | 6.74 | 0.000 |
| Cingulate_Mid_Right | 6 | -24 | 28 | 7.30 | 6.11 | 0.000 |
| Cerebelum_Left | -2 | -62 | -22 | 3.10 | 2.96 | 0.009 |
|  | -6 | -72 | -20 | 2.97 | 2.85 | 0.013 |
| Cerebelum_Right | 8 | -74 | -20 | 2.72 | 2.62 | 0.023 |
| Postcentral_Left | -60 | -20 | 26 | 2.62 | 2.54 | 0.028 |
| Calcarine_Right | 14 | -78 | 10 | 2.40 | 2.34 | 0.045 |
|  | 10 | -82 | 6 | 2.37 | 2.31 | 0.048 |

**Supplementary Table 2**. Activated brain regions associated with reactive inhibition during the stop-signal fMRI experiment. Group level statistical map was calculated as a contrast between successful “switch” trials and successful “go” trials. The results were thresholded at peak-level FDR-corrected significance of p < 0.05.

| Region | MNI coordinates | | | Peak-level | | |
| --- | --- | --- | --- | --- | --- | --- |
|  | x (mm) | y (mm) | z (mm) | T | Z | q_FDR-corr_ |
| Inferior frontal gyrus_Right | 42 | 14 | 28 | 4.20 | 3.49 | 0.005 |
|  | 42 | 8 | 34 | 3.65 | 3.14 | 0.013 |
|  | 48 | 10 | 18 | 3.25 | 2.86 | 0.027 |
|  | 42 | 32 | 18 | 3.43 | 2.99 | 0.019 |
| Supplementary motor area_Left | -6 | -6 | 54 | 3.80 | 3.24 | 0.010 |
|  | -10 | -22 | 48 | 3.24 | 2.86 | 0.027 |
|  | -16 | -18 | 50 | 3.12 | 2.77 | 0.033 |
| Primary motor cortex_Left | -30 | -12 | 66 | 7.13 | 4.92 | 0.000 |
|  | -52 | 6 | 34 | 3.71 | 3.18 | 0.012 |
|  | -48 | 6 | 26 | 3.51 | 3.05 | 0.017 |
|  | -34 | 6 | 24 | 3.18 | 2.81 | 0.030 |
| Pallidum_Left | -12 | -14 | -8 | 3.88 | 3.29 | 0.008 |
| Occipital corext_Left | -8 | -86 | -2 | 13.80 | 6.68 | 0.000 |
| Occipital corext_Right | 14 | -82 | 4 | 11.34 | 6.17 | 0.000 |
|  | 8 | -64 | 0 | 9.87 | 5.80 | 0.000 |
| Postcentral_Left | -38 | -32 | 50 | 6.96 | 4.85 | 0.000 |
|  | -36 | -22 | 54 | 6.72 | 4.75 | 0.000 |
| Hippocampus_Left | -22 | -26 | -6 | 4.92 | 3.90 | 0.001 |
| Cerebelum_Left | -2 | -32 | -4 | 4.30 | 3.55 | 0.004 |
| Frontal_Mid_Left | -40 | 34 | 8 | 3.18 | 2.81 | 0.030 |
| Parietal_Inf_Right | 34 | -50 | 48 | 3.17 | 2.81 | 0.030 |
| Occipital_Sup_Right | 32 | -64 | 40 | 3.12 | 2.77 | 0.033 |
| Angular_Right | 32 | -56 | 42 | 3.10 | 2.75 | 0.035 |
| Thalamus_Right | 6 | -22 | -6 | 3.00 | 2.68 | 0.041 |
| Parietal_Sup_Right | 32 | -56 | 48 | 2.92 | 2.62 | 0.047 |
